# Supplementary material for: Effects of fasting on isolated murine skeletal muscle contractile function during acute hypoxia
Source: PLoS One. 2020 Apr 23;15(4):e0225922. doi: 10.1371/journal.pone.0225922 (PMC7179920; doi:10.1371/journal.pone.0225922)
Supplement: Attachment — Submitted filename: Response to Reviewers.docx [file pone.0225922.s001.docx]

Dear Dr. Mitchell,

We thank you for your time and consideration of our manuscript for publication in Plos One. The reviewers raised many excellent points, and we believe that addressing those concerns has strengthened the quality of the manuscript. Our individual responses to each reviewer’s concerns are addressed below in bold text in the order received.

With Kind Regard,

Joseph M. McClung, PhD

Additional Editor Comments:

The bar graphs used would be much more informative if individual data points were overlaid to show the true variability in the data. 
**-We thank the editor for this suggestion and agree that the recommended change would improve the manuscript. We have modified all bar graphs accordingly. Figures: 2C-F; 3G-H; and 4A-H.**


Review Comments to the Author

**Reviewer #1**: The main idea of this manuscript is investigating the role of muscle glycogen amount for ischemic muscle function. The authors hypothesized that reduced muscle glycogen would shorten the functional time of ischemic muscle. To test this hypothesis, the authors used fasting to reduce glycogen storage and measured muscle functions with hypoxia condition in vitro. Although the idea was interesting, reduced muscle glycogen did not likely change the muscle functions with/without ischemia (rejected hypothesis). I recommend several changes as below to improve the manuscript.
**-We thank the reviewer for their time and for helping us improve the quality of this manuscript with constructive feedback. We have addressed each comment below and summarized the changes made below each comment.**

Fig 1. Need quantification for each image. Vessel density should be normalized by fiber area. Without quantification, this figure can be a supplemental figure. Analyzed fiber number and total animal number should be added to the legend. Make more connections between figure 1 and rest of figures. Otherwise, this figure is little bit out of place.
**-We agree with this assessment and thank the reviewer for this suggestion. In order to keep the manuscript message focused, we have removed the original Figure 1 and replaced it with a schematic diagram that summarizes the experiments (Also addressed in the second comment). We have also removed the paragraph that referred to the original figure 1 from the discussion:**

**“The dynamic requirements of ATP during muscle contraction require a similarly dynamic supply of carbon fuel sources that are derived from both blood and intracellular stores. Physiological and anatomical adaptations (i.e. capillary density, size of stored energy substrate pools, and mitochondrial density/function) are known to facilitate large differences in capacity for spontaneous vs. sustained exercise in different species(44). Similar adaptive differences are highlighted in the distinct anatomical, mechanical, and thermodynamic properties of mouse locomotory EDL and postural soleus muscles(23,27,45,46). These adaptive differences, combined with their similar size and relatively homogeneous fiber type compositions(28), make these muscles excellent candidates for comparative studies of muscle energy metabolism.”**

Fig 2. To help readers better understand this and later figures, Please add scheme (cartoon) of experiment, which will help to understand this and later figures. Also add statistical analysis method for this figure to the legend. Since data have more than 2 factors (O2 vs N2 and Fed vs. Fasting), 2-way ANOVA should be used. (Please note, the Methods section only mentioned a 2 tail student test for group comparison) (Minor comments for Fig 2C-F, adding crosshatch as a fill for the fasting graph (but keeping the color) would make the graph easier to read.)
**-We apologize for this omission. We have updated the statistical analysis methods section to include details of the 2-way ANOVA. We have updated the methods section:**

**Lines: 238-248 “Data are represented by mean ± sample standard deviation (SD). Analyses and plotting were carried out using Graphpad prism (V8.01; Windows 10). Two-way analysis of variance (ANOVA) was used for comparison of group means. Assumption of equal variance was tested using a Brown-Forsythe test. Multiple comparisons were tested using Sidak’s method. Time series data were compared by fitting the curves for each group with an appropriate regression model (simple linear model for O2 conditions; 3-parameter logistic growth model for N2 conditions). Standard deviation of the residuals was used to determine goodness of fit for all curve fitting. Slopes (linear models) and P50 values (i.e. the number of contractions to 50% of the initial force/work; non-linear models) were compared between fed/fasted groups using a sum of squares F-test. P values of < 0.05 were considered statistically significant for all analyses”.**

**Additionally, we have added crosshatch patterns to the fasted group in the bar graphs as requested. Additionally, we have included the individual data points on the bar graphs to more clearly represent the sample variability.**

Fig 3. Add statistical analysis (such as * marks) for each figure and add statistical analysis method (2 way ANOVA is recommended) to the legend. (Same minor comments for Fig 3G-H, adding crosshatch to the fasting graph would improve clarity of the image)
**-We have updated the legends in all figure panels to include details of the relevant statistical analysis. For example: Figure 3 legend:**

**Lines 340-349 “N=8/treatment/group (EDL), N=7/treatment/group soleus. Data are presented as mean ± SD. For the O_2_ condition data, the slope of each line was determined using a simple linear regression model. For N_2_ data, the number of contractions to 50% initial force/work (P_50_) was estimated using non-linear regression. Parameter values (A-D) were compared using a sum of squares F-test. Solid black bars = Fed Group. Crosshatched bars = Fasted Group. Group means (E-H) were compared using two-way ANOVA. ****p<.0001 statistically significant effect of bath aeration condition. ns = no significant effect of feeding condition. # p<.05 statistically significant effect of feeding condition; #### p<.0001 statistically significant effect of feeding condition (Sidak’s multiple comparison test)”.**

Fig 4. Consider change the way the figure is presented. It would be nice combine ATP, ADP, AMP values of all conditions in each single graph like Fig 4G-H. Also add statistical analysis in graph and methods to the legend.
**-We agree that the recommended changes would improve the interpretation of the figure. We have reorganized the graphs in Figure 4 accordingly. Additionally, we have added individual data points to the bar graphs in the manuscript to better represent sample variability. Finally, we have updated the figure legend to include description of the statistical analysis and relevant symbols indicating statistical significance to the graphs.**

Fig 5. Add quantification graph for image analysis, including number of sections and number of mice with statistical analysis method.
**-We understand the reviewer’s concern that the images are not quantified. The images in Figure 5 are intended to demonstrate that muscle is not becoming significantly damaged during the experiment. The images were intended to be qualitative, but thorough in that they demonstrate a lack of damage using multiple histological methods. To address this concern, we have updated the figure legend to highlight the qualitative nature of the images as well as the number of muscles used:**

**Lines: 409-410, 418 “Fig 5: Qualitative assessment of structural integrity of the muscles following experimental protocols.” “N=1/timepoint”).**

**Additionally, we have clarified this point in the results to avoid any confusion:**

**Lines: 406-407 “Together these qualitative assessments did not reveal any indication of damage”).**

Result. Line 319-320. The author claims that “the muscles from the fasted group experienced more rapid reduction in both TTI and work”. However, Fig 3c and 3d did not support this claim due to lack of statistical analysis. Error bars of fed and fasted group graph seem to overlap each other at almost every time point. Please provide detailed support/explanation for this claim.
**-Excellent point, we apologize for any apparent over-interpretation of the data. We have added additional analysis to include a more direct measure of the rate of muscle mechanical failure for statistical comparison. In brief, we performed linear regression for O_2_ data sets and non-linear regression for N_2_ data sets. We selected a three-parameter logistic growth model for the N_2_ data sets, because it gave a similar fit (determined by standard deviation of the residuals) compared to a four-parameter model but required fewer model assumptions. We then compared curve parameters between groups using a sum of squares F-test. The parameters were: slope (for O_2_ groups) and P_50_ (for N_2_ groups; P_50_= number of contractions or time to reach 50% of the initial force/work value predicted by the model).**

**Please note that these additional analyses did not result in any major changes to our previous interpretation of the data. In summary, our conclusion is that fasted groups did reach 50% of initial force/work faster than fed groups. However, the effect of feeding condition was negligible from a practical perspective because it only separated the groups by a matter of minutes.**

**We have highlighted our interpretation of our observations in the results:**

**Lines 316-320 “Both sets of curves were characterized by an inverse linear relationship under O2 conditions and a distinctly non-linear inverse relationship under N2 conditions during the time and frequency domains of the experiments. The muscles from the fasted group experienced more rapid reduction in both TTI and work (Fig A-D)”.**

**We have updated the methods section to include description of the additional analysis:**

**Lines 242-248 “Time series data were compared by fitting the curves for each group with an appropriate regression model (simple linear model for O2 conditions; 3-parameter logistic growth model for N2 conditions). Standard deviation of the residuals was used to determine goodness of fit for all curve fitting. Slopes (linear models) and P50 (i.e. the number of contractions to 50% of the initial force/work; non-linear models) were compared using a sum of squares F-test.”. We have updated Figure 3, the legend, and the methods section to reflect these changes.**

**Reviewer #2:** This manuscript studies the effects of fasting and hypoxia in ex vivo soleus and EDL mouse muscles that are stimulated to contract. The measurements include contractile function, passive tension and metabolites such as glycogen, IMP, and TAN. The premise is that the hypoxia, which is produced by incubated muscles in solution gassed with N2, is a model for ischemia. This premise if flawed. Fasting is used as an intervention to reduce tissue carbohydrate supply. Fasting does lower carbohydrate content of muscles, but that is not the only thing that fasting does. The text needs to be revised to use more direct and accurate language to describe the experimental approach. The major metabolic measurement is glycogen, and some of the values disagree with literature values, which undermines confidence in the data.
**-We thank the reviewer for their time and careful consideration of our manuscript. The provided comments have been very helpful in improving the quality of the work. We have provided individual responses to the comments below, and included brief summaries of the changes made as well as line information that links with the manuscript.**

MAJOR
The title is misleading and should be revised to something more accurate, such as “Effects of fasting and ex vivo hypoxia on murine skeletal muscle contractile function.” The authors don’t have to use this exact wording, but “ischemia” should not be used, “hypoxia” should be used, and the function should be specified.
**-We agree with this concern and have revised the title and main body text to reflect these changes:**

**New title: Lines 1-2 “Effects of fasting on isolated murine skeletal muscle contractile function during acute hypoxia”.**

**Lines: 26-27 Example of use in revised text: “Whether these constraints affect overall functional capacity or the timing of muscle energetic failure during acute hypoxia is not known”.**

Ischemia refers to low blood flow. The muscles are studied ex vivo without any flow, whether they are oxygenated or not. Low oxygen is not the only consequence of ischemia. There is no convincing evidence that this is a good model for ischemia. The repeated use of the word “ischemia” or “ischemic” to describe the experiment should be eliminated. The experiment is studying hypoxia, not ischemia, and the text in the entire manuscript should be revised accordingly. Eliminating or at least deemphasizing the assertion that the experimental approach is an ischemia model would be helpful. If the authors are determined to comment on how this model has relevance to ischemia, they need to provide specific and direct evidence to support this assertion, and to also directly acknowledge the limitations of this experimental approach as an ischemia model.
**-We have revised the text to avoid any misinterpretation of the data by changing the word ‘ischemia’ to ‘hypoxia’ where it refers directly to the model or its interpretation. Example of use in the text:**

**Example Lines78-80 “We hypothesized that fasting, and associated reductions in stored muscle glycogen, would significantly shorten the amount of time that the muscles could remain functional during acute hypoxia”.**

The abstract refers to “conditions of reduced carbohydrate supply” before using a more informative description of “fasting.” The abstract should identify the duration of fasting. The text throughout should also not suggest that all fasting does is reduce carbohydrate supply or glycogen levels. It is OK to indicate that this might be an important consequence of fasting for the effects on contraction function, but there should be a more accurate description of what fasting represents and recognition that reducing glycogen is not everything it does.
**-Excellent point. We have revised the abstract to reflect these changes.**

**Lines: 27-32 “We interrogated skeletal muscle contractile properties in two anatomically distinct hindlimb muscles that have well characterized differences in energetic efficiency (locomotory- extensor digitorum longus (EDL) and postural- soleus muscles) following a 24-hour fasting period that resulted in substantially reduced muscle carbohydrate supply”.**

**Additional lines: 79-81 “We hypothesized that fasting, and associated reductions in stored muscle glycogen, would significantly shorten the amount of time that the muscles could remain functional during acute hypoxia”.**

A major point made by the authors is that glycogen concentration is much higher in the soleus than the EDL. This result has not been observed in earlier research. Glycogen of soleus was not much greater for mouse soleus compared to EDL (Jorgensen J Biol Chem. 2004. 279(2):1070-9; Bonen J Appl Physiol 1994. 76(4):1753-8). The authors should address what might account for the discrepant results and provide evidence that their results are consistent with results of a number of earlier studies.
**-We thank the reviewer for this suggestion, and agree that additional support from the literature will strengthen the interpretation of our data. We have added several additional references to the manuscript:**

- **Ryder JW, Kawano Y, Galuska D, Fahlman R, Wallberg-Henriksson H, Charron MJ, et al. Postexercise glucose uptake and glycogen synthesis in skeletal muscle from GLUT4-deficient mice. FASEB J. 1999;13(15):2246–56.**
- **Sandström ME, Abbate F, Andersson DC, Zhang SJ, Westerblad H, Katz A. Insulin-independent glycogen supercompensation in isolated mouse skeletal muscle: Role of phosphorylase inactivation. Pflugers Arch Eur J Physiol. 2004;448(5):533–8.**
- **Jørgensen SB, Viollet B, Andreelli F, Frøsig C, Birk JB, Schjerling P, et al. Knockout of the α2 but Not α1, 5′-AMP-activated Protein Kinase Isoform Abolishes 5-Aminoimidazole-4-carboxamide-1-β-4-ribofuranoside- but Not Contraction-induced Glucose Uptake in Skeletal Muscle. J Biol Chem. 2004;279(2):1070–9.**
- **Hunter RW, Treebak JT, Wojtaszewski JFP, Sakamoto K. Molecular mechanism by which AMP-activated protein kinase activation promotes glycogen accumulation in muscle. Diabetes. 2011;60(3):766–74.**
- **Azpiazu I, Manchester J, Skurat A V., Roach PJ, Lawrence JC. Control of glycogen synthesis is shared between glucose transport and glycogen synthase in skeletal muscle fibers. Am J Physiol - Endocrinol Metab. 2000;278(2 41-2):234–43.**
- **Helander I, Westerblad H, Katz A. Effects of glucose on contractile function, [Ca 2+] i, and glycogen in isolated mouse skeletal muscle. Am J Physiol - Cell Physiol. 2002;282(6 51-6):1306–12.**
- **Bonen A, Mcdermott J, Tan M. Glycogenesis and Glyconeogenesis in Skeletal: Effects of pH and Hormones. Am J Physiol - Endocrinol Metab. 1990;258(4):693–700.**
- **Bonen A, Homonko DA. Effects of exercise and glycogen depletion on glyconeogenesis in muscle. J Appl Physiol. 1994;76(4):1753–8.**

**We have also modified the discussion to include the following paragraph:**

**Lines: 436-457 “Overnight fasting in rodents results in more dramatic metabolic effects than human overnight fasting, but induces experimentally reproduceable reductions in systemic carbohydrate stores that are similar to more extreme physiological conditions such as hyperinsulinemia, hypoglycemia, or post exercise recovery(11,14,29,39). Fasting was used in this study because it is independent of the confounding effects of exercise or contraction induced fatigue(30). There is a range of reported glycogen values available in the literature for mouse skeletal muscle and liver tissues which are likely influenced by assay method, normalization factor, as well as genetic background and physiological state of the animals(40–45). The fed state values observed for mouse soleus and EDL muscles in this study fall well within the range of normal variability observed in the literature(40–47). Additionally, the magnitude of tissue glycogen reduction observed with 24-hour fasting were concomitant with other available data(29)”.**

The muscle glycogen concentrations are higher than usually reported for mouse EDL and soleus. The value in Table 1 for fed soleus (61.9 nmol/mg) is very high compared to the literature. There should be citations of literature values for glycogen and an explanation for the high values in this study compared to the literature.
**-Addressed in response to the previous comment above. We agree that the values may be interpreted as high compared to some studies, but there are other studies available that report even higher values. Ultimately the absolute values reported in the literature are likely very sensitive to physiological (e.g. insulin/glucagon stimulation, stress responses, diurnal cycle, etc.) and technical variables (e.g. assay method, normalization factor, etc.). To address this concern, we have added the references indicated above to highlight the range of values observed in other studies, and modified the language in our discussion to reflect the variability of reported absolute values:**

**Lines: 441-447 “There is a range of reported glycogen values available in the literature for mouse skeletal muscle and liver tissues which are likely influenced by assay method, normalization factor, as well as genetic background and physiological state of the animals(40–45). The fed state values observed for mouse soleus and EDL muscles in this study fall well within the range of normal variability observed in the literature(40–47). Additionally, the magnitude of tissue glycogen reduction observed with 24-hour fasting were concomitant with other available data(29)”.**

The light/dark cycle times should be stated, and the times when fasting began and when muscles were sampled should be stated.

**-Excellent suggestion. We apologize for this oversight. We have revised the methods section to reflect these changes:**

**Lines: 141-146 “Mice were housed in a temperature-controlled facility on a 12-hour light dark cycle with free access to food and water prior to fasting. Mice were fasted for 24 hours to achieve a reduction of skeletal muscle glycogen of ~50% (compared to the fed state). The 24-hour fasting period began at the beginning of a light cycle and was terminated at the end of the subsequent dark cycle. Mice had free access to water during fasting. Muscles were isolated for experiments immediately following the end of the fasting period.”.**

The Methods section (lines 148-150) on fasting refers to a pilot study and cites a study (ref 23) that is not from this group of authors. It is confusing to know if the authors performed a pilot study or not, and why they cited this study.

**- We apologize for the confusing way that this was presented. We modified the methods section to clarify the procedure:**

**Lines: 141-146 “Mice were housed in a temperature-controlled facility on a 12-hour light dark cycle with free access to food and water prior to fasting. Mice were fasted for 24 hours to achieve a reduction of skeletal muscle glycogen of ~50% (compared to the fed state). The 24-hour fasting period began at the beginning of a light cycle and was terminated at the end of the subsequent dark cycle. Mice had free access to water during fasting. Muscles were isolated for experiments immediately following the end of the fasting period”.**

In the statistics section (lines 242-243), it stated that both SEM and SD are used with the data. Either one or the other should be used. SD is more informative.
**-We apologize for the confusing way that this was presented in the methods. We have modified the methods section to reflect only use only sample standard deviation:**

**Line: 238 “Data are represented by mean ± sample standard deviation (SD)”.**

The Discussion should acknowledge important limitations of the study. One would be that only one timepoint was studied for metabolite concentrations. Measurements at several timepoints would make the study more informative.
**-This is an excellent point. We have added additional discussion of the limitations of our study design:**

**Lines: 490-502 “There are several important limitations to this study. First, the carbohydrate reduction associated with fasting is not complete, leaving approximately half of the fed state muscle glycogen available during experimental hypoxia. Though this is independent of confounding effects associated with other methods of glycogen depletion(30), there are other effects of fasting that may confound observed outcomes(29). Second, though we were able to assess muscle contractile function in time series, we were only able to assess changes in muscle metabolite levels (e.g. adenine nucleotides, glycogen, etc.) at baseline and after the 180-minute protocols. Additional experimentation is necessary to fully characterize the time-dependent changes in key metabolites during hypoxia. Finally, our experimental system utilizing isolated muscle is highly controlled for the effects of hypoxia but lacks the biological variability and complexity of ischemia. We hope that the observations in this study can be used to inform development of hypotheses that can be further tested using in vivo preclinical models of ischemia”.**

The final sentence of the Introduction is that the “This information …ischemia models.” The Conclusion states that the results are valuable for therapeutic intervention (lines 479 and 488). It is unclear why this information will be valuable for either these ischemia models or for therapeutic interventions. It should be directly stated why this information will be valuable.
**-We apologize for any apparent over-interpretation of the data. We have modified the introduction and discussion to clarify our intent and interpretation:**

**Lines: 86-90 “Our data provide a novel characterization of hypoxic muscle mechanical/energetic failure and paint a detailed picture of the timing of these impairments. This information can be used in conjunction with existing in vivo rodent hindlimb ischemia/reperfusion studies(5,16,17,19) to generate new hypotheses regarding optimal timing of reperfusion or administration of precision therapeutics”.**

**Additional lines: 40-42 “Fasting resulted in greater passive tension development in both muscle types, which may have implications for the design of pre-clinical studies involving optimal timing of reperfusion or administration of precision therapeutics”.**

Figure 4 should include text on the figure itself to indicate which A-F panels are from the O2 treatment and which are from N2 treatment.
**-We apologize for this oversight. We have reorganized the panels in this figure to include the basal, O_2_, and N_2_ conditions within each graph for each nucleotide. The feeding conditions and bath conditions are both indicated within each graph. Figure 4A-H. Additionally, we have added cross-hatch patterns to the fasted group bars to help differentiate between the two levels. Finally, we have included individual data points in order to enhance visualization of the data variability.**

In the Introduction (line 76), it stated that the experiment was intended to determine the “exact temporal nature…”, but only one time point (3 hours) was studied, so this study doesn’t determine the “exact temporal nature” of the results.

**-We thank the reviewer for this insight and appreciate the concern. Though we were only able to measure metabolite levels at the terminal endpoint of the experiments, we interpret the force production and work measurements to be informative of the overall energetic state of the muscle during the time course of the experiments. We have modified the introduction to clarify our intent and interpretation of the limitations of the study:**

**Lines: 76-79 “This led us to examine the <3-hour time domain in this study to better define the timing of muscle functional impairments and associated terminal metabolite changes that occur during acute hypoxia”.**
