# Supplementary material for: Effects of fasting on isolated murine skeletal muscle contractile function during acute hypoxia
Source: PLoS One. 2020 Apr 23;15(4):e0225922. doi: 10.1371/journal.pone.0225922 (PMC7179920; doi:10.1371/journal.pone.0225922)
Supplement: Attachment — Submitted filename: Response to Reviewers.docx [file pone.0225922.s002.docx]

Dear Dr. Mitchell,

We thank you for your time and continued consideration of our manuscript for publication in Plos One. We sincerely apologize for failing to adequately address all the reviewer’s concerns in our first resubmission. The reviewer raised several excellent points, and we believe that addressing those concerns has further strengthened the quality of the manuscript. We have separated, what we believe to be the key concerns from the reviewer’s comments below and addressed each individually. We have clarified the discussion in the manuscript and performed some additional data collection relating to the reproducibility of the glycogen assays for the reviewer’s consideration.

With Kind Regard,

Joseph M. McClung, PhD

Reviewer Comments to the Authors:

Reviewer #2: The authors made a number of revisions that have improved the manuscript. However, they have not adequately addressed issues related to the questions about the glycogen results. The current text is not forthcoming in acknowledging or explaining the discrepant glycogen values in this manuscript compared to the literature. The specific concerns are described below.

The authors’ response did not address the fact that their observation of much higher glycogen in the soleus than the EDL, and this result has not been observed in earlier research. Glycogen of soleus was not much greater for mouse soleus compared to EDL (Jorgensen J Biol Chem. 2004. 279(2):1070-9; Bonen J Appl Physiol 1994. 76(4):1753-8). The authors should address what might account for the discrepant results and provide evidence that their results are consistent with results of a number of earlier studies. The authors cited the 2 publications above (which found soleus values were slightly, but not significantly lower for soleus versus EDL) and 6 additional publications, none of which reported glycogen for both soleus and EDL in mice. The authors did not acknowledge that their results of 80% greater glycogen in soleus compared to EDL are at odds with the published literature, and they offered no data that supported their discrepant results. There needs to be a direct explanation in the manuscript related to the unusual findings in this study.

The value for soleus glycogen in Table 1 (61.9 nmol/mg) is much greater than previously published values. The authors state that other studies have reported even higher values, but they don’t point out which studies reported higher values. In the revised manuscript, they state the observed values “in this study fall well within the range of normal availability observed in the literature”, and they cite 8 publications (#40-47). However, several of these publications do not report mouse soleus or EDL glycogen values, and the studies that do report glycogen in these muscle do not report values higher than 61.9 nmol/mg. Some cited studies only reported gastrocnemius glycogen, or single fiber glycogen, or not glycogen concentrations (only glycogenesis rates). Citations that do not include mouse soleus and or EDL glycogen concentrations should not be cited to support the statement in the manuscript. The text needs to be revised to be accurate. The text refers to values being influenced by “assay method, normalization factor, as well as genetic and physiological state of the animals.” This statement is true, but it doesn’t explain the much greater value in this study. If there is any specific evidence that the genetic or physiological state of the mice, or the particular assay used in this study led to much increased soleus glycogen values, it should be specifically identified rather than this general statement without any direct support of its validity.

Dear Reviewer #2,

We sincerely apologize for failing to appropriately address the concerns regarding the reported muscle glycogen values. The references included in the last resubmission were chosen to highlight a range of potential factors that reflect variation in reported values for mouse skeletal muscle glycogen, but clearly, we missed the mark. We appreciate the reviewer’s concerns and thank them for their ongoing efforts in aiding our improvement of the quality of this manuscript. Below, we attempt to address all the key concerns that we interpret from the provided comments.

1. “The authors should address what might account for the discrepant results”

We have added additional text to the discussion. Lines 448-458. “Though we cannot directly account for specific confounders that explain the discrepancy in this study, there are two likely candidates that should be considered for further investigation: 1.) Muscle glycogen levels have been shown to vary with season and diurnal cycle in mice(44) and rats(45,46), with peaks in the dark-light cycle transition period (the time at which animals were sacrificed in this study). 2.) Soleus muscles have been shown to be more sensitive to insulin stimulated glucose uptake and glycogen synthesis in both mice(38) and rats(47), and insulin stimulated glucoregulatory responses have been shown to differ among inbred mouse strains(48). Taken together, the described findings support the possibility that stored muscle glycogen values may have been influenced by seasonal, circadian, or hormonal variation intrinsic to the genetic background of the mice used in this study”.

2. “provide evidence that their results are consistent with results of a number of earlier studies”

Our observed glycogen values differed from three prior studies that assessed mouse EDL and soleus muscle glycogen content using similar assay methods (cited in the paper and listed below; these were the best matched studies we could find in terms of methodology and including both EDL and soleus muscles). We have altered the manuscript to clearly acknowledge that the results differ from the cited studies and added discussion of potential confounding variables (see response described above). We have found it difficult to directly account for the discrepancies. However, there are a few methodological and environmental differences between this study, and the cited references (particularly contribution of diurnal variation, seasonal variation, and parental genetic background variation as discussed). We have been fully transparent regarding our methods/data analysis and feel that the methodological description in this manuscript is thorough and comparable to all of the published studies that we have reviewed. Additionally, all supporting raw data will be available to the scientific community via open science framework if the manuscript is accepted for publication (in accordance with Plos One’s publishing requirements). We value the importance of ensuring that the data are of high quality and reproducibility. However, we also appreciate that biological data is variable and subject to substantial influence from a multitude of confounding factors. If there are any additional specific methodological concerns, we will enthusiastically address them. We don’t want to risk sharing inaccurate data, but we also want to avoid denying the scientific community access to data solely on the basis that it differs from previous findings.

**Some additional technical considerations:** For brevity, we reported overall sample size for the groups by using the smallest group size (as this has the largest effect on the ANOVA power). The actual sample sizes for the fed state basal EDL and soleus is N=17 and N=10 respectively. This occurred because of additional myography (twitch protocols) and biochemical assays (UPLC amino acid profiles) that ultimately were not included in the manuscript in order to keep the supporting evidence concise. The soleus glycogen data does have two outliers (87.2 and 96.2 nmol/mg) that skew the mean toward a higher value, however, we did not have any reasonable evidence that the values should be excluded from the data set (61.9±17.5nmol/mg with outliers; 54.4±8.6 nmol/mg without outliers; Mean±SD). Even if the outliers were excluded, the soleus glycogen would have been higher than the EDL values (34.4±8.0 nmol/mg), and both were higher than the values reported in the cited references.

We do not have the original lysates used to determine the glycogen values from the original study and re-performing all of the assays would necessitate re-performing the entire study. However, in the interest of ensuring that our assay methods were sound, we did attempt to assess the reproducibility of the assay. For this quality control (QC) assay, we performed additional glycogen measurements in muscles isolated from BALB/c mice. However, the mice that we had available were younger and of the opposite sex than those used in the study, therefore it is difficult to rule out contributions of biological variability from the reproduced assays. We were able to match the circadian timing for sacrifice, but not seasonal timing (Summer for study vs. winter for QC). However, we provide the data for comparison below. Though the higher glycogen values in the soleus observed in the study were not reproduced, we interpret the consistent values for the tibialis anterior muscles to indicate that the assay is sound and reproducible, and that the previously observed discrepancy was likely derived from biological variation rather than technical variation. Some of the difference in values between study and QC values may also be attributable to large differences in muscle size between the two groups. In both cases the samples were run on the same plate, in triplicate, with R^2^ of the standard curve ≥.99.

| Muscle | Sample size (N) | Mean Glycogen (nmol/mg tissue wet weight) | Standard deviation |
| --- | --- | --- | --- |
| Tibialis Anterior (study) | 18 | 38.7 | 6.7 |
| Tibialis Anterior (QC Assay) | 6 | 32.3 | 2.6 |
| EDL (study) | 17 | 34.4 | 8.0 |
| EDL (QC) | 6 | 23.1 | 2.4 |
| Soleus (Study) | 10 | 61.9 | 17.5 |
| Soleus (QC) | 6 | 27.3 | 6.7 |

3. “The authors did not acknowledge that their results of 80% greater glycogen in soleus compared to EDL are at odds with the published literature, and they offered no data that supported their discrepant results”

We have added additional discussion to the manuscript to fully acknowledge the discrepancy and to try to explain potential sources of variability in the data. Lines 436-455 “Overnight fasting in rodents results in more dramatic metabolic effects than human overnight fasting, but induces experimentally reproduceable reductions in systemic carbohydrate stores that are similar to more extreme physiological conditions such as hyperinsulinemia, hypoglycemia, or post exercise recovery(1–4). Fasting was used in this study because it is independent of the confounding effects of exercise or contraction induced fatigue(5). Notably, the glycogen values observed in this study differ from several previous reports in that fed state control values for both muscles are relatively high, and that the soleus glycogen levels are substantially higher than the EDL (values between muscles did not differ in the previous reports)(6–8).

Muscle glycogen concentration is a physiologically dynamic parameter that is influenced by experimental conditions such as assay method and normalization factor, as well as biological conditions such as parental genetic background and metabolic state (9–11). Though we cannot directly account for specific confounders that explain the discrepancy in this study, there are two likely candidates that should be considered for further investigation: 1.) Muscle glycogen levels have been shown to vary with season and diurnal cycle in mice(12) and rats(13,14), with peaks in the dark-light cycle transition period (the time at which animals were sacrificed in this study). 2.) Soleus muscles have been shown to be more sensitive to insulin stimulated glucose uptake and glycogen synthesis in both mice(6) and rats(15), and insulin stimulated glucoregulatory responses have been shown to differ among inbred mouse strains(16). Taken together, the described findings support the possibility that stored muscle glycogen values may have been influenced by seasonal, circadian, or hormonal variation intrinsic the genetic background of the mice used in this study”.

Additional Lines 486-498 “In this study, we observed that Soleus muscles stored more glycogen at baseline, had greater specific force/work capacities, and produced absolute force for a longer period during hypoxia compared to EDL muscles. The observations regarding greater glycogen content in the soleus muscle compared to EDL muscles are not consistent with previous reports(7,8,17), but the observations of improved mechanical function during hypoxia in soleus compared to EDL muscles have been previously reported using small muscles isolated from rats(18). Though the absolute differences in glycogen concentrations between groups were larger in the soleus compared to the EDL, the response coefficient (R_Glyc_) which facilitates interpretation of group differences relative to their baseline concentration, indicated that the patterns of utilization were not different between the two types of muscles. We interpret these findings to mean that the greater basal glycogen concentration observed in the soleus muscles was likely not the primary factor underlying it’s enhanced ischemic mechanical performance“.

4. “The authors state that other studies have reported even higher values, but they don’t point out which studies reported higher values”
   a. We apologize for any confusion regarding our interpretation of the additional references provided in the previous submission. Our intent was to show that the measured values fell within a reasonable range of values relative to previous reports (i.e. that the values were physically possible). We have been careful to ensure that any specific references to published data are clearly linked to relevant text (see above paragraphs and additional references). Additionally, we have removed any references that do not directly reference the EDL and/or Soleus muscles.
5. “Citations that do not include mouse soleus and or EDL glycogen concentrations should not be cited to support the statement in the manuscript.”
   a. We have removed two of the cited references that did not specifically assess glycogen content in mouse EDL and/or soleus muscles.
6. “If there is any specific evidence that the genetic or physiological state of the mice, or the particular assay used in this study led to much increased soleus glycogen values, it should be specifically identified rather than this general statement without any direct support of its validity.”
   a. As discussed, the discrepancies in the measured values are difficult to account for directly. However, there are a few candidate sources of variation that have been more thoroughly discussed in the manuscript in an attempt to account for the differences. These include: references describing seasonal and diurnal variation in muscle glycogen content in mice and rats. Diurnal variation in muscle glycogen typically peaks at the end of the dark cycle/beginning of the light cycle, when the animals in this study were sacrificed. Thus, we think it reasonable to suggest that this may be a confounding variable here. Additionally, soleus muscles in mice and rats have been shown to be more sensitive to insulin stimulated glucose uptake.

 References:

1.	Vollestad NK, Blom C. Effect of varying intensity on glyocgen depletion in human muscle fibres. Acta Physiol Scand. 1985;125:395–405.

2.	Cohen N, Rossetti L, Shlimovich P, Halberstam M, Hu M, Shamoon H. Counterregulation of hypoglycemia: Skeletal muscle glycogen metabolism during three hours of physiological hyperinsulinemia in humans. Diabetes. 1995;44(4):423–30.

3.	Jensen TL, Kiersgaard MK, Sørensen DB, Mikkelsen LF. Fasting of mice: A review. Lab Anim. 2013;47(4):225–40.

4.	Adeva-Andany MM, González-Lucán M, Donapetry-García C, Fernández-Fernández C, Ameneiros-Rodríguez E. Glycogen metabolism in humans. BBA Clin. 2016;5:85–100.

5.	Jennische E. Ischaemia Induced Injury in Glycogen-Depleted Skeletal Muscle. Selective Vulnerability of FG Fibres. Acta Physiol Scandanavia. 1985;125:727–34.

6.	Bonen A, Mcdermott J, Tan M. Glycogenesis and Glyconeogenesis in Skeletal: Effects of pH and Hormones. Am J Physiol - Endocrinol Metab. 1990;258(4):693–700.

7.	Jørgensen SB, Viollet B, Andreelli F, Frøsig C, Birk JB, Schjerling P, et al. Knockout of the α2 but Not α1, 5′-AMP-activated Protein Kinase Isoform Abolishes 5-Aminoimidazole-4-carboxamide-1-β-4-ribofuranoside- but Not Contraction-induced Glucose Uptake in Skeletal Muscle. J Biol Chem. 2004;279(2):1070–9.

8.	Bonen A, Homonko DA. Effects of exercise and glycogen depletion on glyconeogenesis in muscle. J Appl Physiol. 1994;76(4):1753–8.

9.	Sandström ME, Abbate F, Andersson DC, Zhang SJ, Westerblad H, Katz A. Insulin-independent glycogen supercompensation in isolated mouse skeletal muscle: Role of phosphorylase inactivation. Pflugers Arch Eur J Physiol. 2004;448(5):533–8.

10.	Hunter RW, Treebak JT, Wojtaszewski JFP, Sakamoto K. Molecular mechanism by which AMP-activated protein kinase activation promotes glycogen accumulation in muscle. Diabetes. 2011;60(3):766–74.

11.	Helander I, Westerblad H, Katz A. Effects of glucose on contractile function, [Ca 2+] i, and glycogen in isolated mouse skeletal muscle. Am J Physiol - Cell Physiol. 2002;282(6 51-6):1306–12.

12.	Pessacq MT, Gagliardino JJ. Glycogen metabolism in muscle: Its circadian and seasonal variations. Metabolism. 1975;24(6):737–43.

13.	Conlee RK, Rennie MJ, Winder WW. Skeletal muscle glycogen content: diurnal variation and effects of fasting. Am J Physiol. 1976;231(2):614–8.

14.	Funabiki R, Ide S, Ebe N, Taguchi O, Yasuda K. Diurnal changes in glycogen content in skeletal muscle of meal-fed rats during starvation. Agric Biol Chem. 1975;39(4):791–3.

15.	Leighton B, Challiss RAJ, Lozeman FJ, Newsholme EA. Effects of dexamethasone treatment on insulin-stimulated rates of glycolysis and glycogen synthesis in isolated incubated skeletal muscles of the rat. Biochem J. 1987;246(2):551–4.

16.	Berglund ED, Li CY, Poffenberger G, Ayala JE, Fueger PT, Willis SE, et al. Glucose metabolism in vivo in four commonly used inbred mouse strains. Diabetes. 2008;57(7):1790–9.

17.	Hamada T, Arias EB, Cartee GD. Increased submaximal insulin-stimulated glucose uptake in mouse skeletal muscle after treadmill exercise. J Appl Physiol. 2006;101(5):1368–76.

18.	Howlett RA, Hogan MC. Effect of hypoxia on fatigue development in rat muscle composed of different fibre types. Exp Physiol. 2007;92(5):887–94.
