# Supplementary material for: Effects of fasting on isolated murine skeletal muscle contractile function during acute hypoxia
Source: PLoS One. 2020 Apr 23;15(4):e0225922. doi: 10.1371/journal.pone.0225922 (PMC7179920; doi:10.1371/journal.pone.0225922)
Supplement: Attachment — Submitted filename: Response to Reviewers.docx [file pone.0225922.s003.docx]

Dear Dr. Mitchell,

We thank you for your time and continued consideration of our manuscript for publication in Plos One. We have added the additional information to the methods section as indicated in the response below.

With Kind Regard,

Joseph M. McClung, PhD

Reviewer #2: Because circadian and seasonal effects are described as important for glycogen values, please specify for each experiment (if is the same for every experiment, that can be noted and it can be stated only once):

1) The times in the day when lights were turned on and turned off.

2) The times in the day when samples were collected.

3) The month(s) when experiments were performed

We thank the reviewer for their continued time and effort. We appreciate the constructive nature of the feedback and feel that the manuscript has been substantially improved by our exchanges. To address the outlined concerns, we have added additional details to the methods section:

Lines 141-149 “Mice were housed in a temperature-controlled facility on a 12-hour light-dark cycle with free access to food and water prior to fasting (dark cycle: began at 1900 hours, ended at 0700 hours). Mice were fasted for 24 hours to achieve a reduction of skeletal muscle glycogen of ~50%, compared to the fed state. The 24-hour fasting period started at the beginning of a light cycle (0700 hours) and was terminated at the end of the subsequent dark cycle (0700 hours). Mice had free access to water during fasting. All muscles (including control and fasted groups) were isolated for experiments immediately following the end of the dark cycle, between 0700 and 0800 hours. All experiments were performed in the summer season, between the months of May and August”.
